# MULTIPLE MEDICATION USE AND RISK OF TREATED FALL INJURY: THE HEALTH ABC STUDY

**DOI:** 10.1093/geroni/igz038.069

**Published:** 2019-11-08

**Authors:** Lingshu Xue, Robert M Boudreau, Julie M Donohue, Janice C Zgibor, Zachary A Marcum, Tina Costacou, Anne B Newman, Elsa Strotmeyer

**Affiliations:** 1 University of Pittsburgh, Pittsburgh, United States; 2 University of Pittsburgh, Pittsburgh, Pennsylvania, United States; 3 University of South Florida, Tampa, Florida, United States; 4 University of Washington, Seattle, Washington, United States

## Abstract

Multiple medication use within one year is associated with increased fall injury risk in older adults. However, chronically using multiple medications and treated fall injury have rarely been explored, particularly in cohort studies linked with claims data. We examined using >5 medications in 2 or more consecutive years (chronic medication use) as a risk factor for treated fall injury in 1,898 community-dwelling adults (age 73.6±2.9 years; 53% women; 37% black) with linked Medicare Fee-For-Service (FFS) claims from the Health, Aging and Body Composition Study since 1997/98 clinic visit. Incident fall injury (N=546) was the first claim from 1998/99 clinic visit to 12/31/08 with an ICD-9 fall code and non-fracture injury code, or fracture code with/without a fall code. Stepwise Cox models with a time-varying predictor of chronic medication use before fall injury or censoring (N=414) vs. not using >5 medications at the same time (N=1008) were adjusted for baseline demographics, lifestyle factors, fall history, quadriceps strength, cardiovascular disease (CVD), diabetes, sensory nerve impairment, and kidney function. Fall injury risk increased for chronic medication users (37%) vs. non-users (29%) (HR=1.25[1.00-1.57]), though was attenuated after adjustment for CVD and diabetes (HR=1.18[0.93-1.51]). Sensitivity analyses excluding fall-risk-increasing drugs (FRIDs) from medication counts (HR=1.32[0.54-3.20]), or including those using >5 medications non-chronically (N=365) in referent groups (HR=1.22[0.96-1.55]) had consistent findings. Unmeasured comorbidity differences may confound associations of chronic medication use and treated fall injury risk in older adults with Medicare FFS. Considering both chronic diseases and medication use in fall risk assessments is needed.

